# The Effect of Poly (Methyl Methacrylate) Content on Chemical, Thermomechanical, Mechanical, and Fatigue Life Characteristics of Ternary PC/ABS/PMMA Blends

**DOI:** 10.3390/polym17141905

**Published:** 2025-07-10

**Authors:** Hamdi Kuleyin, Recep Gümrük

**Affiliations:** 1 Department of Mechanical Engineering, Recep Tayyip Erdoğan University, 53100 Rize, Türkiye; 2 Department of Mechanical Engineering, Karadeniz Technical University, 61080 Trabzon, Türkiye; rgumruk@ktu.edu.tr

**Keywords:** ternary blends, characterization, mechanical behavior, fatigue life, thermomechanical behavior, polymer blends, pc/abs, pmma

## Abstract

Polymer blending techniques enable the tailoring of desired properties for diverse applications. This study investigates the effect of PMMA content on the thermomechanical, chemical, mechanical, and fatigue life properties of PC/ABS/PMMA (polycarbonate/acrylonitrile–butadiene–styrene/polymethylmethacrylate) ternary blends. To this end, various characterization analyses, as well as tensile, impact, and fatigue tests, were conducted. The results indicate that the viscoelastic modulus improves with increasing PMMA content in ternary blends. Furthermore, PC/ABS/PMMA blends exhibit an immiscible phase morphology. The elastic modulus, yield strength, and tensile strength increase with higher PMMA content, while the elongation at break and impact strength decrease. Fatigue strength and the fatigue strength exponent were found to vary nonlinearly with PMMA content. Compared to PC/ABS blends, PC/ABS/PMMA blends demonstrated improvements of approximately 12% to 58% and 26% to 117% in hysteresis energy and the dynamic elastic modulus, respectively. Additionally, fatigue life cycles improved by 5% to 11% at low stress amplitudes. This experimental study provides comprehensive insight into the complex interplay among the chemical, thermomechanical, mechanical, and fatigue properties of ternary PC/ABS/PMMA blends, highlighting their potential for applications requiring balanced or tailored structural and material characteristics.

## 1. Introduction

The blending of two or more polymers is a widely adopted technique for achieving balanced and tailored material properties—such as thermal stability, impact strength, rigidity, and chemical resistance—for a range of industries, including the automotive, aerospace, transportation, drug delivery, biomedical application, and electronic device industries [1,2,3,4]. The advantages of obtaining polymer blends by melt blending include optimizing desired properties specific to applications or parts, reducing production costs, increasing production flexibility, and improving production process efficiency [5,6]. Therefore, the polymer blending method has attracted intense interest in both academic and industrial fields due to its advantages during material production, such as obtaining the desired or balanced properties, recyclability, and waste management strategies [7,8].

One of the most popular polymer blends, the polycarbonate (PC) and acrylonitrile–butadiene–styrene (ABS) blend, has been widely used in automotive, aerospace, and electronics applications [9]. In this blend, the PC/ABS combination stands out in these application areas due to its advantageous properties derived from both PC (which offers high impact resistance, thermal stability, and rigidity) and ABS (which provides excellent processability, toughness, and low costs) [10]. Despite their wide range of applications and the advantages they offer, PC/ABS blends also have certain limitations, including UV sensitivity, flammability, limited heat resistance, and poor wear and chemical resistance [11,12]. To overcome limitations such as poor mechanical and thermal properties and the wear, flammability, and water resistance of PC/ABS blends, methods for creating new PC/ABS-based blend structures using compatibilizers, fillers, agents, or polymers are frequently used in the literature [13,14,15,16]. For this purpose, ongoing research focuses on enhancing the usability of PC/ABS-based blend structures in various industrial applications. The incorporation of a third or more polymer components into the PC/ABS system aims to tailor the blends for specific properties or application requirements. A considerable number of studies have been devoted to exploring various compatibilization strategies to improve the overall performance of PC/ABS blends. For instance, Zhang et al. [17] determined that the addition of an ABS copolymer grafted with maleic anhydride (ABS-g-MAH) as a compatibilizer in the PC/ABS blend matrix had a notable effect on the impact strength of the blends, although it had a limited effect on their tensile properties. Similarly, the effects of compatibilizers such as MAH-grafted polypropylene, EVA-g-MAH, and MAH-grafted styrene–ethylene/butylene–styrene (SEBS-g-MAH) on the compatibility of PC/ABS blends were investigated [18,19,20,21]. Verma et al. [22] examined the influence of a styrene–isoprene–styrene (SIS) copolymer on the 3D printability and mechanical properties of PC/ABS blends. They concluded that SIS improved compatibility between the components and the printability of the blends; additionally, both the impact strength and elongation-at-break values increased depending on the SIS content in the blend system. Dai et al. [23] investigated the effects of maleic anhydride-grafted ABS (ABS-g-MAH) and methyl methacrylate–butadiene–styrene (MBS) compatibilizers on the tensile, impact, and microstructural properties of PC/ABS blends. It was concluded that while the distribution of the compatibilizer within the PC and SAN phases may enhance interfacial adhesion, it does not serve a toughening function in terms of impact strength. In addition to improving compatibilization between PC and ABS, Hong et al. used PCL as a compatibilizer and examined the effects of PCL content on the thermal, morphological, mechanical, and viscoelastic properties of PC/ABS/PCL ternary blends [24]. It was demonstrated that the addition of PCL to PC/ABS improved compatibility as well as enhancing the mechanical properties. Seo et al. [9] investigated the influence of the blend ratio on the rheological and mechanical properties of PC/ABS/PBD blends. In addition, the effects of the number of recycling cycles on the mechanical and material properties of injection-molded PC/ABS-based ternary blends with thermoplastic polymers such as PA6, PMMA, and PBT were examined through experimental studies [25,26,27]. These findings highlighted the recyclability characteristics of PC/ABS blends in terms of polymer waste management and their mechanical, chemical, rheological, thermal, and physical properties.

Numerous studies in the literature have been focused on improving the physical, rheological, mechanical, thermal, and compatibility properties of PC/ABS blends by incorporating brittle polymers such as polystyrene (PS), styrene/acrylonitrile (SAN) copolymers, poly(methyl methacrylate) (PMMA), acrylonitrile styrene acrylate (ASA), and methyl methacrylate–butadiene–styrene (MBS) [28,29,30]. Among these materials, PC/ABS blends modified with PMMA have come to the forefront due to their ability to significantly reduce the ABS particle size within the matrix, improve the interfacial adhesion between polymer components, enhance mechanical properties, reduce notch sensitivity, and modify the fracture mechanism [29,31,32]. Liu and Bertilson [33] and Rybnicek [26] reported that the addition of PMMA to PC/SAN and PC/ABS blends significantly improved phase adhesion, resulting in enhanced compatibility, miscibility, and elongation properties. Most recently, Ezzeddine et al. [11] investigated the mechanical and physical properties of recycled PC/ABS/PMMA blends produced at different composition ratios from end-of-life computer plastics. They identified the optimal blend ratio for achieving balanced mechanical and physical properties based on both experimental results and ANOVA modeling. PMMA has been widely used in the literature for similar purposes, either to form binary blends or as a third component in ternary systems. For example, Moussaif and Jérôme examined the effect of PMMA addition on the structural compatibility, phase morphology, and mechanical properties of an immiscible PC/PVDF blend [34]. As a result of the study, it was stated that both the phase distribution and mechanical properties were improved with the addition of 20% PMMA. Bubmann et al. [35] obtained PC/PMMA binary blends through reactive compounding and reported enhanced phase adhesion behavior and the formation of a uniformly distributed phase structure with superior mechanical properties.

Numerous studies on PC/ABS polymer blends have focused on their thermal, mechanical, impact, morphological, and rheological properties [9,28,36,37]. The relationships between the content of polymer components, additives, or fillers and these properties have also been examined. However, studies on PC/ABS/PMMA ternary blends in the literature have primarily focused on the interactions among physical, morphological, and mechanical properties [11,25,38]. This study aims to investigate the effect of PMMA content on the thermal, chemical, thermomechanical, mechanical, and fatigue life properties of PC/ABS/PMMA ternary blends. To achieve this, several characterization analyses and tests were conducted. The study intends to contribute to the development of polymer blends with balanced performance, particularly for potential applications in automotive components, the aerospace industry, and consumer goods.

## 2. Materials and Methods

### 2.1. Materials and the Production of Specimens

In this study, granule raw materials were selected as the polycarbonate (PC)/acrylonitrile–butadiene–styrene (ABS) and poly (methyl methacrylate) for specimen preparation due to their common usage in various industrial products. PC granules, Makrolon^®^ AL2447, were obtained from Covestro Company; ABS granules, commercially available Starex^®^SR-0325LT, were supplied by Lotte Chemical Company (Seoul, Republic of Korea); and PMMA granules, from the Sumipex^®^ME automotive-grade series, were also supplied from Lotte Chemical Company. The physical and mechanical properties of the supplied granules (PC, ABS, and PMMA) are presented in Table 1.

Before the injection-molding process, the granules were dried at 90 °C for 4 h to prevent moisture-related effects and were physically mixed according to the desired composition ratios of the injection-molded parts. After mixing, the granule premixes were fed into the barrel. Injection molding was carried out using a fully automatic Haitian 160 series injection-molding machine equipped with twin-screw extrusion. To avoid undesirable geometric defects, shrinkage, and ejector pin marks on the specimen gauge area or gauge surface during direct specimen geometry injection, the injection-molding process was completed using flat plate geometry. Injection-molded PC/ABS, PMMA, and PC/ABS/PMMA ternary blend specimens were produced as flat plates measuring 150 mm × 150 mm by the melt blending method from dried and physically mixed granules. During the injection-molding process, the parameters of the PC/ABS, PMMA, and PC/ABS/PMMA blends (at different composition ratios) were set as follows: an injection pressure of 14 MPa, a holding pressure of 4 MPa, an injection time of 1.5 s, and a holding time of 3 s. The barrel temperature profile consisted of six zones—zones 1 through 5 and the nozzle (zone 6)—set at 230 °C, 240 °C, 250 °C, 250 °C, 255 °C, and 245 °C, respectively. Additionally, the screw speed was maintained constant at 40 rpm throughout the process.

**Figure 1:**
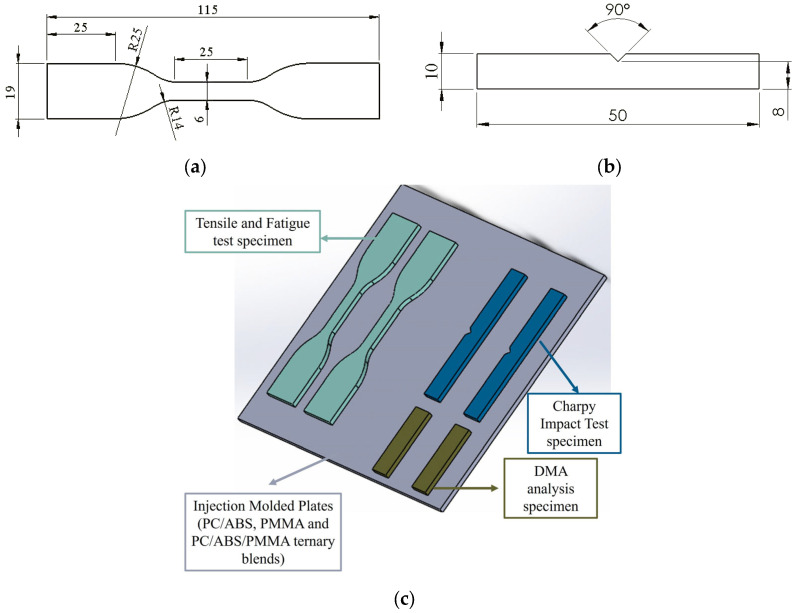
Geometry and dimensions (in mm) of the (**a**) tensile (dog-bone) and fatigue specimens and (**b**) notched impact specimen; and (**c**) schematic illustration of specimen placement on the injection-molded plates.

The PC/ABS binary blends were produced with a ratio of 60% PC and 40% ABS, and the PC/ABS/PMMA ternary blends were prepared with a constant PC/ABS ratio of 60/40 wt.; the content of PMMA was varied in steps by 20, 40, 60, and 80 weight percent. The PC/ABS blend ratio was selected based on the advantageous properties exhibited at this specific composition, including high impact strength, good processability, electro-coating compatibility, balanced thermal and mechanical performance, and elevated heat resistance. Owing to these properties, PC/ABS blends with this ratio are widely utilized as commercial products by global manufacturers particularly in the automotive industry. The blend flat plates were categorized according to their composition ratios as PC/ABS_20, PC/ABS_40, PC/ABS_60, and PC/ABS_80, where the numbers represent the weight percentage of PMMA in the ternary blend (PC/ABS/PMMA) structure. Since the PC/ABS ratio remained constant in the ternary blend specimens and to make the classification clearer, the notation was defined as “PC/ABS_(PMMA content wt.)”. The test specimens for the tensile and dynamic mechanical analysis and the impact and fatigue tests were obtained from flat plates using a water jet cutting method to prevent any thermal effects. A schematic illustrating specimen replacement on injection-molded flat plates for water jet cutting is given in Figure 1c.

### 2.2. Analyses and Testing Procedures

This section presents detailed information about the analysis and testing procedures to determine the chemical, thermomechanical, mechanical, and fatigue life characteristics of the PMMA, PC/ABS, and ternary blend specimens.

Fourier Transform Infrared Spectroscopy (FTIR) analysis was performed to determine the chemical structure. This analysis was conducted with attenuated total reflection (ATR) diamond crystal modules on the Perkin Elmer 100 series IR spectrometer in the wavenumber range of 4000–500 cm^−1^ at a resolution of 2 cm^−1^, with 16 scans performed to reduce the noise. To evaluate the miscibility of the blend system, Differential Scanning Calorimetry (DSC) analysis was performed. The glass transition temperatures of the neat and blended specimens were obtained from DSC analysis, providing key information about the miscibility characteristics of the blends. DSC analyses were carried out under a nitrogen flow of 20 mL/min within a temperature range of 25–200 °C, employing two heating and cooling cycles at a temperature increase rate of 10 °C/min. The thermomechanical properties of the neat and blended specimens were determined via dynamic mechanical analysis (DMA), conducted in accordance with the ASTM D5023 standard [42]. These analyses were completed in temperature sweep mode with a nitrogen gas flow of 80 mL/min, a temperature range of 25–200 °C, a 1 Hz frequency, and a 1 °C/min heating rate. DMA specimens, with 40 mm × 10 mm × 3 mm dimensions, were extracted from the flat plates. Additionally, following ASTM D5023 recommendations, the specimens were conditioned at 23 °C and 50 ± 10% relative humidity prior to analysis.

Tensile tests were performed to determine the mechanical properties of the specimens, including the elastic modulus, yield strength, ultimate tensile strength, and elongation at break, as well as to examine the effects of PMMA content on these properties. The tensile tests were conducted according to the ASTM D638 standard [43] using type 4 specimen geometry (Figure 1a). A BESMAK servo-hydraulic universal test machine equipped with a 25 kN load cell was used, and the tests were completed at a 5 mm/min crosshead velocity at room temperature following ASTM D638 recommendations. The elongation in the gauge section was measured using a strain gauge extensometer. Also, to ensure repeatability, at least five tensile tests were performed in accordance with ASTM D638. To evaluate the effect of PMMA content on the impact strength of the PC/ABS/PMMA ternary blends, Charpy notched impact tests were conducted with an Instron Ceast 9050 impact tester (capacity of 50 J) in accordance with ISO 179 standard [41]. Impact test specimens with dimensions of 3 × 10 × 50 mm with a centered V-notch and a length of 2 mm were obtained with water jet cutting from injection-molded flat plates (Figure 1b). The impact tests were completed by taking at least three measurements, and the average values were recorded. The impact strength was calculated using the following equation:

Impact Strength (J/m^2^) = E/A (1)

where E is the measured energy of the fracture for the specimens and A is the area of the cross section.

To determine the fatigue life behavior of the PMMA, PC/ABS, and ternary blend specimens, stress-controlled fatigue tests were performed at a constant stress ratio (S_max_/S_min_ (R) = 0.1) and at a test frequency of 5 Hz in accordance with the ASTM D7791 standard [44]. Fatigue tests were conducted using type 4 specimen geometry (ASTM D638) in accordance with the ASTM D7791 standard. The tests were carried out on a BESMAK servo-hydraulic testing machine equipped with a 25 kN load cell, similarly to the tensile tests. The tests were performed in a sinusoidal loading scenario under tensile/tensile loading, and a stress–strain hysteresis curve was obtained every 50 cycles with dynamically controlled software. Fatigue tests were completed following the standard (ASTM D7791), with at least four different stress amplitudes and by performing at least three repeated fatigue tests to ensure the repeatability of the tests (minimum of twelve specimens for each test).

The fatigue life characteristics of the PMMA, PC/ABS, and PC/ABS/PMMA ternary blend specimens, as well as the effect of PMMA content on fatigue life behavior, were evaluated by plotting the number of cycles to failure against stress amplitude as S-N curves. To analyze the fatigue life characteristics, the well-known Basquin equation (Equation (2)), a semi-empirical model, was used to predict the fatigue life of the materials:

S = A(N)^b^ (2)

where S is the stress amplitude, N is the number of cycles to failure at each stress amplitude, A is the fatigue stress coefficient, and b is the fatigue stress exponent.

## 3. Results

This section presents the experimental findings on the chemical, thermal, viscoelastic, mechanical, and fatigue life properties of PC/ABS, PMMA, and PC/ABS/PMMA ternary blends at different composition ratios. With these findings, the fundamental relationship between the chemical and viscoelastic properties and the mechanical characteristics was assessed, considering the effects of PMMA content. The results obtained from characterization analyses and mechanical tests are presented in the following subsections.

### 3.1. Chemical Properties

**Figure 2:**
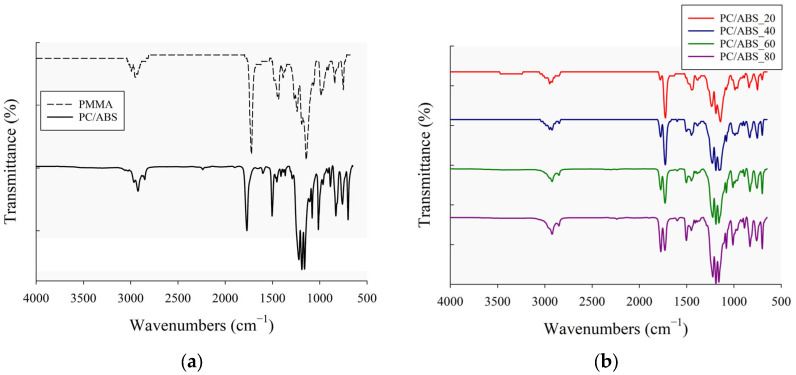
The obtained IR spectra from FTIR analysis: (**a**) PMMA and PC/ABS materials, and (**b**) PC/ABS/PMMA ternary blend materials at different composition ratios.

FTIR analysis provided crucial information about the chemical bonds, interactions, absorption peaks, and wavenumbers of the PMMA, PC/ABS and PC/ABS/PMMA ternary blends. For detailed examination, the IR spectra of the PMMA and PC/ABS materials were analyzed to identify their characteristic peaks. Subsequently, the IR spectra obtained from the blend structure were evaluated by a comparison with those of the neat components. Furthermore, possible changes in peak intensity, shifts in wavenumbers, and the formation of new peaks were investigated. The IR spectra of the PMMA, PC/ABS, and ternary blends are presented in Figure 2.

The IR spectra of the granule materials, namely PMMA and PC/ABS, are given in Figure 2a. The characteristic peaks corresponding to the chemical bond structures of the materials can be identified in the figure. For PMMA, the peaks observed at wavenumbers 2990 cm^−1^, 2950 cm^−1^, 1435 cm^−1^, and 1140 cm^−1^ are linked with C-H strength vibrations, CH stretching, methyl/methylene group bending vibrations (C-H), and O=C-O ester bond stretching vibrations, respectively [45,46]. The peak at wavenumber 1719 cm^−1^ corresponds to the C=O (carbonyl) stretching vibration band [45], which is characteristic of the ester group in PMMA. This vibration band peak also represents the bonding interactions within the ternary blend system between the hydrogen groups of PMMA and ABS [47,48]. To evaluate basic chemical bonds in the ternary blend system, the PC/ABS IR spectrum was examined in detail. For the PC/ABS material containing 60% PC and 40%ABS, the IR spectrum includes characteristic peaks originating from both the polycarbonate and acrylonitrile–butadiene–styrene components. The spectrum shows PC-related chemical bond vibrations at 1766 cm^−1^ and 1011 cm^−1^, corresponding to the molecular stretching of -C=O, and peaks at 1220 cm^−1^, 1190 cm^−1^, and 1153 cm^−1^, attributed to the C-O stretching vibration bands [49,50]. Also, at wavenumbers 752–700 cm^−1^, the phenyl ring bands associated with C-H bending from the polycarbonate chemical structure appear, as well as the absorption band of the methyl group in the wavenumber range of 3000–2800 cm^−1^ [49]. In Figure 2a, the peaks at 2238 cm^−1^, 1600 cm^−1^, and 910 cm^−1^ correspond to the nitrile group (C≡N) from AN in the ABS, C=C stretching, and =C-H bending bands, respectively, which are characteristic peaks of ABS [51,52]. Peaks associated with styrene-phase stretching are observed at wavenumbers 1500 cm^−1^ and 1600 cm^−1^ [53]. The peaks at wavenumbers 964 cm^−1^ and 910 cm^−1^ are representative of C-H bending, resulting from hydrogen atom interactions in the butadiene phase in ABS [54]. Overall, the presence of peaks at 2238 cm^−1^, 1766 cm^−1^, 1011 cm^−1^, 964 cm^−1^, and 910 cm^−1^ confirms that the blend under analysis consists of PC and ABS polymer components.

The IR spectra obtained from FTIR analysis of the ternary blend specimens of different compositions are shown in Figure 2b. From this figure, no new or different peaks are observed in these spectra other than the characteristic peaks of the PC, ABS, and PMMA components. All the absorption bands correspond to the known structural groups of these three compounds. In the ternary blend structure, the peaks at 2238 cm^−1^, 1719 cm^−1^, 1260–1140 cm^−1^, and 750–700 cm^−1^ are associated with the nitrile group (ABS), ester carbonyl group (PMMA and PC), C-O strength (PMMA), and aromatic rings (PC/ABS), respectively. However, it is observed that only the characteristic peak intensities tend to increase or decrease according to the PMMA or PC/ABS composition ratio in the ternary blend structure. For example, the characteristic peaks resulting from PMMA in the ternary blends at 1719 cm^−1^ and 1435 cm^−1^ tend to decrease in intensity with decreasing PMMA content. Similarly, the peaks at 2238 cm^−1^, 1766 cm^−1^, and 1260–1140 cm^−1^ tend to increase in intensity with the increasing PC/ABS content in the ternary blends. Furthermore, the absorption band intensity observed at 1719 cm^−1^ increases with higher PMMA composition ratios, resulting in high-intensity peaks in ternary blends containing 60% and 80% PMMA. These high-intensity peaks suggest that the blend system may form a miscible phase within the structure [55]. Table 2 summarizes the characteristic observed peaks for the ternary blend specimens and their corresponding raw materials.

### 3.2. Thermal Properties

**Figure 3:**
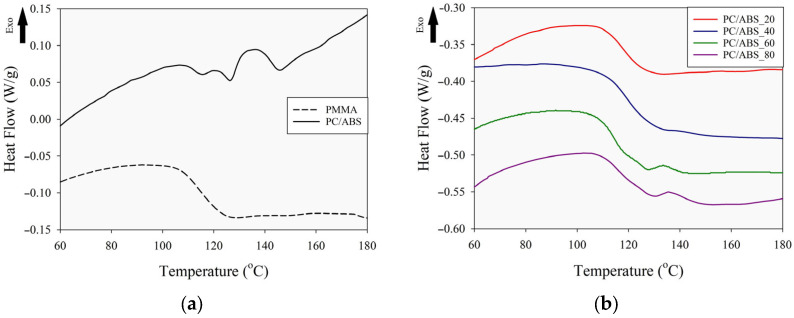
DSC thermogram (second heating) of the specimens: (**a**) PMMA and PC/ABS; (**b**) PC/ABS/PMMA ternary blends at different compositions.

For the determination of the miscibility behavior of the blend structures, the glass transition temperatures of the specimens were determined with DSC analysis. Glass transition temperatures provide insight into the miscibility characteristics of blend materials; for example, a single transition temperature indicates a miscible structure, whereas two or more transition points correspond to an immiscible formation. The heat flow versus temperature curves (DSC thermograms) obtained from the second heating–cooling cycles of the DSC analysis of the PMMA, PC/ABS, and PC/ABS/PMMA ternary blends are given in Figure 3. The glass transition temperature values were taken from the second heating scan to eliminate the effects of prior thermal history during the melt blending and injection-molding processes. In the figure, a step-like decrease region in the heat flow is observed in the temperature range of 100–150 °C for the specimens. It can be said that the change in heat flow as a step revealed the transition temperature region in the DSC thermograms. To determine the exact T_g_ values, second derivatives of the heat flow–temperature curves were calculated as a function of temperature, and the resulting T_g_ values are summarized in Table 3.

From Figure 3a, the glass transition temperature of PMMA was determined to be approximately 114 °C. In the case of PC/ABS, more than one step-like decrease can be observed in the heat flow versus temperature curve. This phenomenon is commonly encountered in two-component amorphous blends. Accordingly, three different glass transition temperatures were identified at approximately 113 °C, 125 °C, and 147 °C. The glass transition temperature at 147 °C is well-defined and corresponds to the transition region associated with the polycarbonate component. The relatively small distinct transition region observed in the range of 110–130 °C represents the transition region originating from ABS. In addition, it can be stated that the transition region originating from the PC component of the PC/ABS blend structure is more prominent and exhibits a more dominant character due to the higher PC component ratio in the blend.

The heat flow variations with respect to the temperature of the ternary blend specimens, obtained from DSC analysis, are given in Figure 3b. In the figure, the heat flow changes based on the temperature show different characteristics according to the PMMA content in the ternary blends. For the PC/ABS_20 ternary blend, a distinct single-step decrease in heat flow was obtained in the 110–130 °C range. This region indicates that the blend exhibits a miscible formation due to the presence of a single glass transition temperature. However, with decreasing PMMA content in the ternary blend system (i.e., for the PC/ABS_40 and PC/ABS_60 specimens), the heat flow curves exhibited two step decreases in the 100–150 °C temperature range. Additionally, the PC/ABS_80 blend sample showed a three-step decrease in the heat flow curve, indicating that immiscible phase formation occurs in these compositions. As seen in Table 3 and Figure 3, with the increase in PC/ABS content in the ternary blend system, glass temperature values originating from the PC and ABS components are observed. This behavior is particularly evident in the PC/ABS_60 and PC/ABS_80 specimens. Moreover, the temperature at which the glass transition of the polycarbonate phase is observed tends to increase with the rising PC/ABS ratio. Glass transition temperatures originating from all the components forming the ternary blend structure were observed in the PC/ABS_80 specimen. In the specimens with lower PC/ABS content, although the transition region attributed to the PC phase was detected, a distinct decrease in heat flow corresponding to the ABS component was not clearly observed. This is likely due to the fact that the T_g1_ value (representing the glass transition temperature of the styrene–acrylonitrile phase) in the PC/ABS sample is very close to the glass transition temperature of the PMMA component. Similarly, it can be said that the heat flow step decreasing region (which relates to the butadiene phase) originating from the ABS component and observed at approximately 126 °C in PC/ABS does not form a distinctive region at low PC/ABS composition ratios in ternary blends.

### 3.3. Thermomechanical Properties

**Figure 4:**
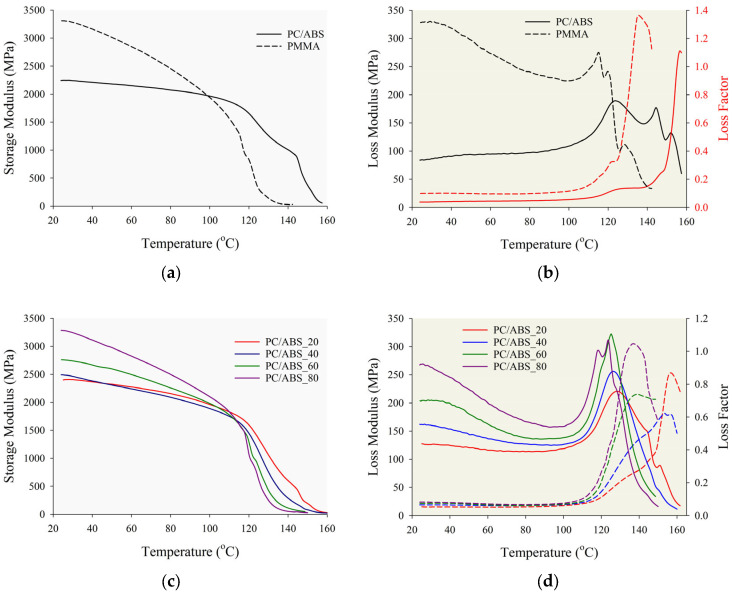
Viscoelastic properties obtained from DMA analysis. (**a**) Storage modulus of PMMA and PC/ABS; (**b**) loss modulus and loss factors of PMMA and PC/ABS; (**c**) storage modulus of PC/ABS/PMMA ternary blends; (**d**) loss modulus and loss factors of PC/ABS/PMMA ternary blends.

The thermomechanical properties, such as the storage modulus, loss modulus, and loss factors, of the PMMA, PC/ABS, and PC/ABS/PMMA ternary blends were examined to evaluate the viscoelastic behavior of the specimens with respect to their composition ratios. Also, the findings obtained from DMA analysis were used to explain the mechanical and fatigue behavior of the specimens. The DMA analysis results are presented in Figure 4 and Table 4.

Figure 4a,c presents the storage modulus versus temperature curves for the PMMA, PC/ABS, and PC/ABS/PMMA ternary blends with different contents. The storage modulus is a key mechanical parameter that indicates a material’s ability to store elastic energy and, thus, its stiffness [56]. A detailed examination of the storage modulus–temperature curves in Figure 4a reveals that PMMA exhibits a notably high initial storage modulus of approximately 3309 MPa. In contrast, the PC/ABS sample shows a significantly lower storage modulus of around 2245 MPa with respect to PMMA. At the same time, a sharp decrease in the storage modulus is observed for PMMA as the temperature increases, especially in the 90–120 °C range. This temperature range corresponds to the glass transition temperature region of PMMA, and in this region, the storage modulus value approaches zero, with a sudden decrease. This decrease is associated with the increased mobility of the polymer chains and approaching the glass transition temperature. In other words, the load-carrying capacity of the material decreases significantly. However, PMMA has a very high initial stiffness at approximately room temperature, i.e., a superior load-carrying capacity. However, the sudden decrease in the storage modulus also indicates that the thermal stability of the structure is low. For the PC/ABS sample, the storage modulus decreases more gradually with temperature, suggesting that it maintains stiffness over a broader temperature range than PMMA. Thus, it can be said that the PC/ABS sample has superior thermal stability and impact strength. The storage modulus versus temperature curves for the PC/ABS/PMMA ternary blend specimens were examined, and in Figure 4c, it is observed that the storage modulus especially increases with the increasing PMMA composition ratio in the blend structure at low temperatures. For example, at room temperature, the PC/ABS_20 sample has a storage modulus of approximately 2340 MPa, whereas the PC/ABS_60 and PC/ABS_80 samples reach approximately 2760 MPa and 3280 MPa, respectively. Furthermore, the temperature-dependence decline in the storage modulus varies with PMMA content. It is observed that the slope of the decreasing storage modulus with temperature increases with the increasing PMMA composition ratio in the structure (Table 4). It can be said that the temperature-dependent storage modulus changes of the ternary blend samples containing a lower PMMA ratio exhibit a more stable behavior. Based on storage modulus data, it can be concluded that at low PMMA ratios, the PC/ABS matrix phase dominates the behavior, while increasing PMMA content enhances the initial stiffness but compromises thermal stability.

It is well known that the loss modulus represents the extent to which a material exhibits viscous behavior. As shown in Figure 4b, the loss modulus of the PMMA and PC/ABS specimens decreases sharply after reaching its peak in the glass transition region of PMMA. In this region, the energy absorption capacity significantly diminishes due to the softening of the amorphous phase. For the PC/ABS specimen, it can be seen that the loss modulus value shows a steady increase with temperature up to the peak point, and after this point, which represents the glass transition region, the decrease follows a slower trend than for PMMA. At lower temperatures (particularly at the initial stage), PMMA exhibits a considerably higher loss modulus value (328 MPa) than PC/ABS (84 MPa). This behavior is associated with the greater energy absorption capacity and the more viscous nature of PMMA. This behavior is associated with the greater energy absorption capacity and the more viscous nature of the PMMA. In a similar manner, an examination of the loss modulus curves presented in Figure 4d for the PC/ABS/PMMA ternary blend specimens reveals that both the initial and peak loss modulus values increase in proportion to the rising PMMA ratio within the blend. This indicates that as the PMMA increases, the loss modulus increases, and the structure becomes more viscous in form. When the loss factor curves are examined, it is seen that PMMA has a higher peak value than PC/ABS (Figure 4b). At the same time, it was observed that the peak value of the loss factor increased with the increasing PMMA ratio in the ternary blend specimens. Also, the DMA results showed that blends with a higher PMMA content had a larger area under the loss modulus curve, reflecting greater structural energy absorption, while lower PMMA content resulted in a smaller area under the loss modulus curve. This finding suggests that the energy absorption capability of the material improves with increasing PMMA content. However, it can also be inferred that a more brittle structure with high internal friction is formed, particularly in the PC/ABS_80 sample, where the highest peak value was observed.

### 3.4. Mechanical Properties

**Figure 5:**
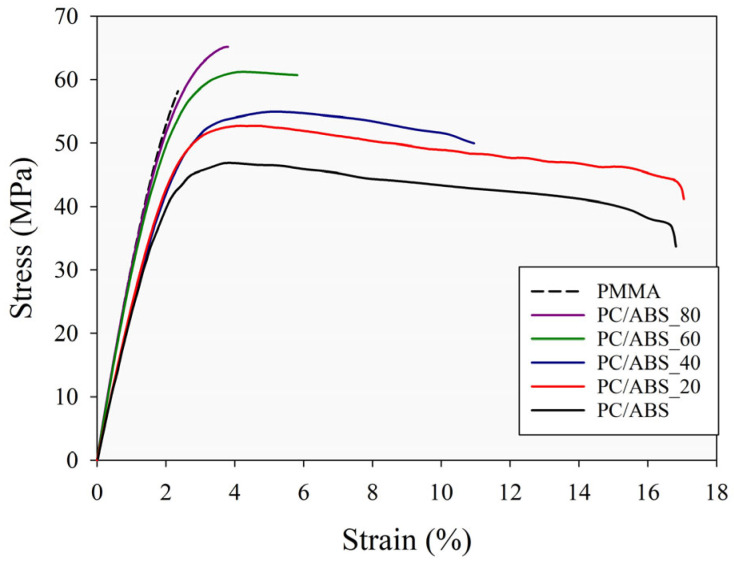
Stress–strain curves obtained from the tensile tests for PMMA, PC/ABS, and PC/ABS/PMMA ternary blends.

Mechanical properties such as the elastic modulus, yield strength, ultimate tensile strength, and elongation at break were obtained from the tensile tests of the PMMA, PC/ABS, and PC/ABS/PMMA ternary blends at different composition ratios. The effect of the composition ratios on the mechanical properties and impact strength were investigated, and the stress–strain curves of the specimens and the summarized mechanical properties of the specimens are given in Figure 5 and Table 5, respectively.

Figure 5 and Table 5 show that the PMMA specimen exhibits brittle characteristic behavior, as indicated by it having the lowest elongation at break. However, the highest elastic modulus value was obtained from this specimen. In contrast, the PC/ABS specimen displayed significantly lower values for the elastic modulus, yield strength, and ultimate tensile strength than PMMA while exhibiting a considerably higher elongation at break (Table 5). Also, the ternary blend specimens exhibited higher elastic modulus, yield strength, and ultimate tensile strength values than the PC/ABS specimen. In the ternary blend samples, it was observed that with the increasing PMMA ratio in the ternary blend structure, the elasticity modulus, yield strength, and tensile strength values increased, while the elongation-at-break values decreased. For instance, with increasing PMMA content, the elastic modulus of the ternary blends improved by 1%, 6%, 30%, and 37% for 20, 40, 60, and 80 wt. of PMMA addition, respectively, compared to PC/ABS. Similarly, it was determined that the ternary specimens PC/ABS_20, PC/ABS_40, PC/ABS_60, and PC/ABS_80 increased their yield strengths by 8%, 13%, 19%, and 27%, respectively, and their ultimate tensile strengths by 13%, 17%, 33%, and 41%, respectively, relative to the PC/ABS specimen. In this context, it can be concluded that the improvements in mechanical properties, except for the elongation at break, exhibit an approximately linear trend with increasing PMMA content. Likewise, the reduction in the elongation at break follows a linear trend as the PMMA ratio increases in the ternary blend.

When evaluated within the framework of tensile test results, it was determined that the ternary blend specimens have superior mechanical properties to the PC/ABS specimen, especially in terms of yield strength, tensile strength, and modulus of elasticity properties. In similar studies in the literature, it has been stated that the increase in yield strength with an increasing PMMA ratio in the structure is due to the high degree of compatibility of the blend structure [25]. This situation is explained by the stress transfer between the phases when sufficient interfacial adhesion is achieved [38]. It can be said that the PC/ABS_20 and PC/ABS_40 specimens, in particular, exhibit a competitive character compared to the PC/ABS specimen in terms of the mechanical properties (when the elongation at break is also considered). Also, the elongation at break decreases upon addition of more than 20% PMMA, which clearly leads to reduced ductility of the blend compared to PC/ABS. However, for ternary blends, even when there is a high composition of PMMA, good impact toughness is maintained thanks to the toughening effects results from PC/ABS [57,58] (Table 5).

In addition, when the impact strengths given in Table 5 are examined, it can be determined that PMMA has low impact strength (approximately 3.8 J/m^2^) as a result of its brittle behavior. On the other hand, the PC/ABS specimen has high impact strength (82.4 J/m^2^) as expected from the results obtained from the tensile tests and viscoelastic properties. In the PC/ABS/PMMA ternary blend samples, it is observed that the impact strength decreases significantly with the increasing PMMA content in the structure. A similar situation is also seen in previous studies in the literature for PMMA-based blends [11,36]. When the mechanical behavior of the ternary blends is evaluated together with their thermal stability and viscoelastic properties, it can be said that this will be an important alternative for obtaining materials with desired mechanical properties based on different application areas, such as rigidity, toughness, impact strength, and fracture mechanisms. Thus, it can be concluded that the ternary blend (PC/ABS/PMMA) specimens obtained by the melt blending method can be optimized in a way that exhibits balanced properties.

### 3.5. Fatigue Life Behavior

**Figure 6:**
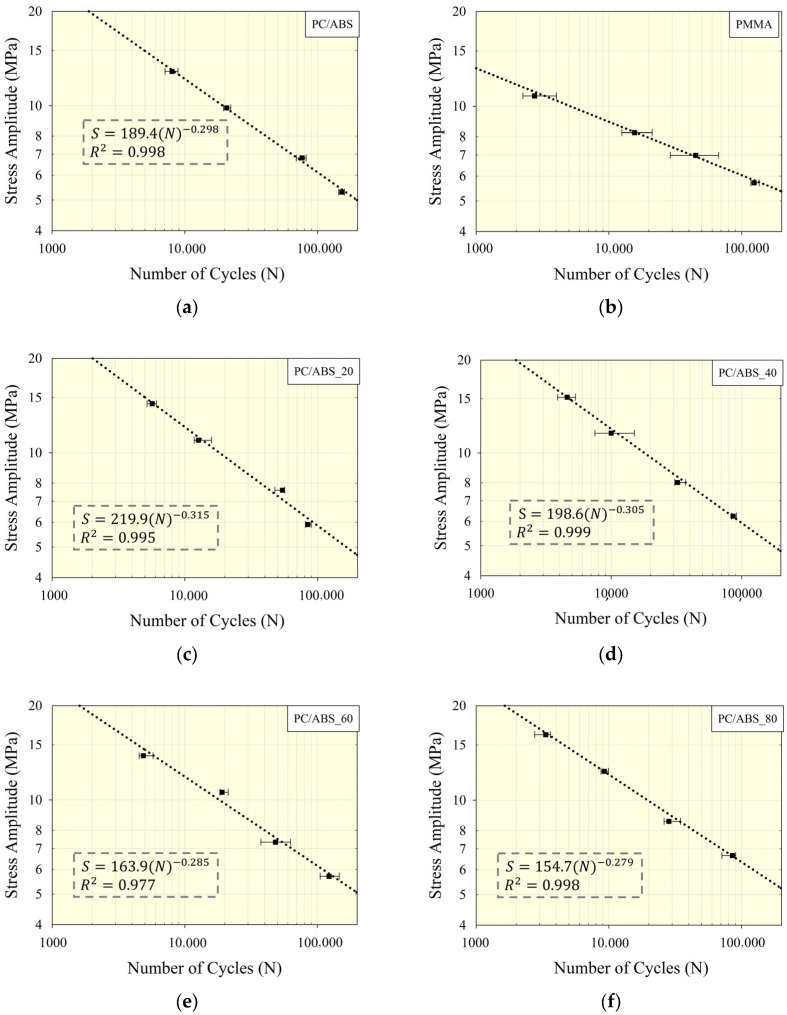
The number of cycles to failure and S-N curves obtained from fatigue tests and the Basquin equation: (**a**) PC/ABS; (**b**) PMMA; (**c**) PC/ABS_20 blend; (**d**) PC/ABS_40 blend; (**e**) PC/ABS_60 blend, (**f**) PC/ABS_80 blend.

The effect of PMMA composition on the fatigue life characteristics of the PC/ABS/PMMA ternary blends was examined through uniaxial fatigue tests. Figure 6 presents the stress amplitude versus number of cycles (S-N) curves for the PMMA, PC/ABS, and PC/ABS/PMMA ternary blends at different composition ratios. In addition, the fatigue strength and fatigue strength exponent, derived from these tests using the Basquin equation, characterizing fatigue life behavior, are shown on the curves. In addition to this, the experimental data obtained from the fatigue tests are summarized in Table 6. To better understand the influence of the composition ratio, changes in the coefficients of the Basquin equation were analyzed in detail.

When the S-N curves in Figure 6 and the cycle numbers summarized in Table 6 are considered, it is seen that PMMA has a lower fatigue strength (~39.5 MPa) and fatigue strength exponent (absolute value = 0.174). This situation indicates that PMMA exhibits low fatigue strength, and at the same time, the cycle number value is represented by Basquin coefficients, with a lower slope depending on the stress amplitude. It was determined that PC/ABS has superior fatigue strength and a higher absolute fatigue strength exponent value compared to PMMA. Thus, it is easily understood that PC/ABS will fail at the end of a higher number of cycles than PMMA at similar stress amplitude values. In the ternary blend specimens, it was determined that the change in fatigue strength and fatigue strength exponent values with the addition of PMMA to the structure occurred nonlinearly (Figure 6). While it was observed that the strength and absolute exponent values increased with the addition of 20% PMMA, fatigue strength and the absolute exponent coefficients decreased with the addition of 40, 60, and 80% PMMA compared to the addition of 20% PMMA. The fatigue strength exponent (b) expresses how the fatigue life changes with the change in stress amplitude. For higher PMMA content (60 and 80%), it has been determined that both the fatigue strength and the absolute exponent value show a decreasing trend compared to the coefficients for the PC/ABS specimen, indicating that fatigue life performances are improved at similar stress amplitudes.

From the fatigue strength exponents given in Figure 6, especially the observed decrease in the absolute values compared to PC/ABS, it can be concluded that as stress amplitudes decrease, the fatigue life performance improves for the PC/ABS_60 and PC/ABS_80 specimens. In addition, the fatigue strength exponent of the PMMA specimen is lower than those of the other specimens, which corresponds to a decrease in fatigue life with decreasing stress amplitude. It was also determined that fatigue strength values in the PC/ABS/PMMA ternary blend samples showed a nonlinear variation with PMMA content, initially increasing and then decreasing. This suggests that blends containing relatively low PMMA content (20% and 40%) have superior strength per cycle, meaning that they may fail at higher stress amplitudes and lower cycle numbers (≤10,000 cycles). The change in fatigue strength depending on the PMMA ratio in the ternary blend can be said to arise from increased miscibility and structural compatibility in structures containing 20% and 40% PMMA (Section 3.2 and Section 3.3).

## 4. Discussion

**Figure 7:**
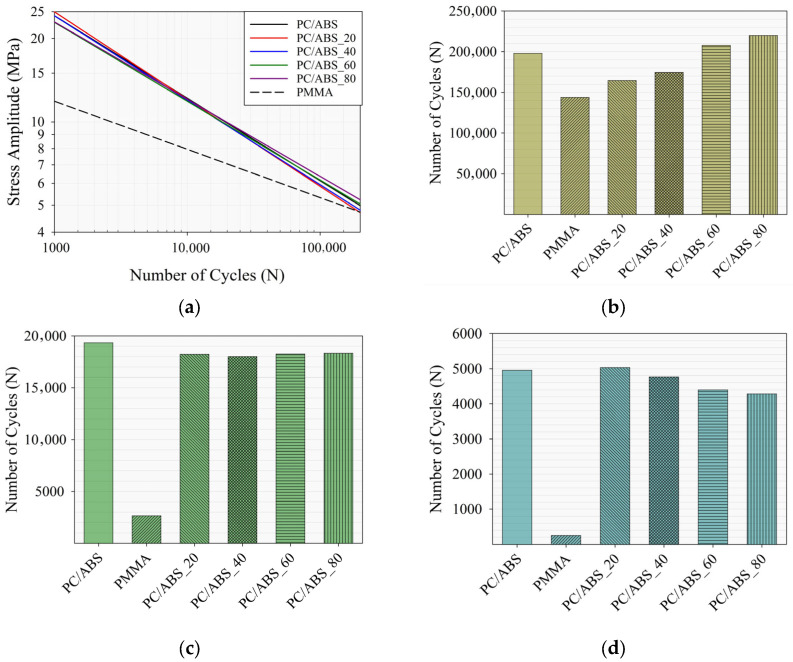
(**a**) S-N in comparable form obtained with Basquin coefficients, and the number of cycles for specimens at stress amplitudes of (**b**) 5 MPa, (**c**) 10 MPa, and (**d**) 15 MPa.

The effect of PMMA content on the fatigue life characteristics of the PC/ABS/PMMA ternary blends were examined in detail, and the curves obtained via the Basquin equation coefficients for the specimens are presented comparatively in Figure 7a. In addition, changes in the number of cycles to failure determined at the same stress amplitudes, depending on the composition ratio, were calculated using the Basquin equation and are given comparatively for different stress amplitudes (5, 10, and 15 MPa) in Figure 7b–d, respectively. The effects of the coefficients obtained from the Basquin equation on the number of cycles at low and high stress amplitude values are illustrated through these curves (Figure 7b–d).

It is known that fatigue strength represents the maximum stress value in the first cycle, while the fatigue strength exponent represents the increasing (or decreasing) trend of the number of cycles according to the stress amplitude. Therefore, the coefficients obtained from the Basquin equation are critically important for examining the fatigue behavior of blend structures with different composition ratios. For this purpose, changes in the coefficients for PC/ABS with the addition of PMMA to the blend were examined in terms of cycle numbers at low and high stress amplitudes. It can be said that samples with a high fatigue strength coefficient have higher stress amplitude values at lower cycle numbers. For example, it can be said that the PC/ABS_20 and PC/ABS_40 samples with superior fatigue strength coefficients compared to PC/ABS samples will fail (~1000 cycle) at higher stress amplitude values at the same cycle number. At the same time, it can be concluded that the PC/ABS_60 and PC/ABS_80 specimens, which have higher absolute fatigue strength exponent values than PC/ABS, will fail after a higher number of cycles at the same low stress amplitudes (≤10 MPa). This trend is demonstrated by the coefficients of the specimens at stress amplitude values of 5, 10, and 15 MPa and the corresponding number of cycles predicted by the Basquin equation (Equation (2)) (Figure 7b–d). For instance, it was determined that the PC/ABS_20 sample showed superior fatigue life performance to PC/ABS at a high stress amplitude value of 15 MPa due to its high fatigue strength value. It was also determined from the Basquin coefficients that at a 15 MPa stress amplitude, the PC/ABS and PMMA samples would fail after approximately 4961 and 261 cycles, respectively, whereas the PC/ABS_20 sample would fail after 5035 cycles. In addition, it was observed that for a 15 MPa stress amplitude, the fatigue life cycle performance was competitive with that of PC/ABS with an increasing PMMA ratio. For example, the PC/ABS_40, PC/ABS_60, and PC/ABS_80 samples were determined to fail after 4768, 4404, and 4288 cycles, respectively, using Basquin coefficients. On the other hand, it was determined that the fatigue strength exponent decreased in absolute value compared to the PC/ABS sample, and in the PC/ABS_60 and PC/ABS_80 samples, the number of cycles increased at low stress amplitude values. For example, it was determined that at a 5 MPa stress amplitude, PC/ABS would fail after approximately 198,000 cycles, whereas the PC/ABS_60 and PC/ABS_80 specimens would fail after approximately 208,000 and 220,000 cycles, respectively. For a 5 MPa stress amplitude, it was determined that the ternary blend specimens improved their failure cycle numbers by 5 and 11 percent, respectively, compared to PC/ABS. At a 10 MPa stress amplitude, it was observed that the PC/ABS and PC/ABS/PMMA ternary blend samples containing PMMA at different composition ratios exhibited similar fatigue life performances. This stress amplitude can be considered the transition region of fatigue life behavior at low and high stress amplitudes and the intermediate stress amplitude region for these materials (Figure 7b).

To quantify the fatigue life characteristics and their changes with PMMA content in the PC/ABS/PMMA ternary blends, the parameters from the hysteresis curves and dynamic modulus were determined. The hysteresis loops were assessed as hysteresis areas, defined by the difference between the integrals of the loading and unloading curves. Also, the dynamic modulus of elasticity (E_dyn_) was calculated as follows [58]:

(3) $$E_{dyn}=\frac{\sigma_{max}-\sigma_{min}}{\varepsilon_{max}-\varepsilon_{min}}$$

**Figure 8:**
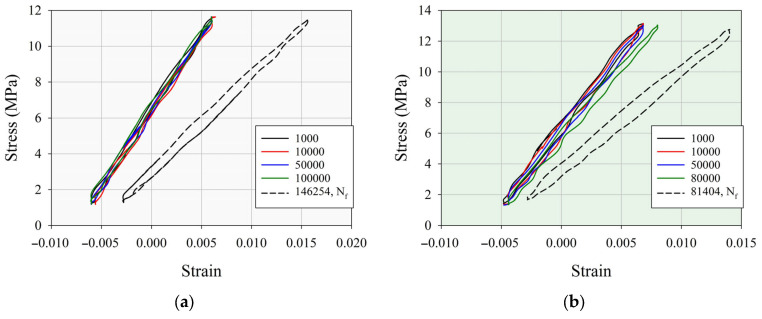

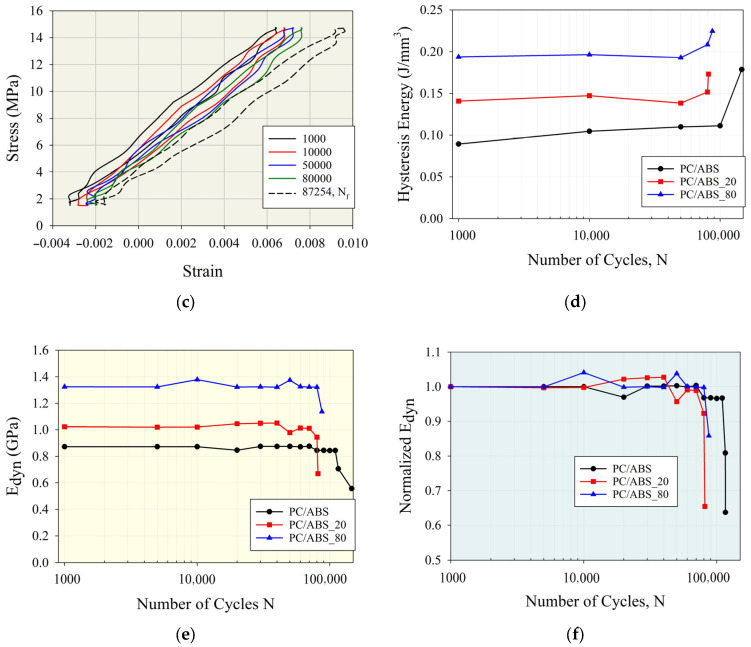
Stress–strain hysteresis curves at different cycles: (**a**) PC/ABS, (**b**) PC/ABS_20, (**c**) PC/ABS_80 blend; and compatible curves depending on cyclic loading obtained from hysteresis loops: (**d**) hysteresis energy, (**e**) dynamic elastic modulus, (**f)** normalized dynamic elastic modulus.

The hysteresis loops and hysteresis energies (hysteresis areas) were determined for the PC/ABS, PCABS_20, and PCABS_80 specimens at low stress amplitudes, such as 5.3 MPa, 5.9 MPa, and 6.7 MPa, respectively, obtained from the fatigue tests. These specimens fractured after 146,254, 81,404, and 87,254 cycles, respectively. The stress–strain hysteresis curves and hysteresis energy, dynamic elastic modulus, and normalized dynamic elastic modulus versus number of cycles curves are presented in Figure 8.

From the hysteresis stress–strain curves given in Figure 8a–c, the loop shifts depending on the cycle loading during the fatigue tests can be examined. To assess the loop shifts seen in the hysteresis curves, for the PC/ABS, PC/ABS_20, and PC/ABS_80 specimens, the stress–strain hysteresis curves obtained at the 1000th, 10,000th, and 50,000th cycles were considered, respectively. As seen from the figure for these cycles, the loop shift has a high value for the PC/ABS_80 specimen, representing the plastic deformation or damage accumulation due to cyclic loading of the tension/tension fatigue tests [59,60]. In addition to the hysteresis energies calculated from the stress–strain hysteresis curves given in Figure 8a–c, the changes in hysteresis energies with respect to the number of cycles are given in Figure 8d. From the figure, the highest hysteresis energy value during the loading and unloading stages is obtained for the PC/ABS_80 ternary blend. The 1000th cycle was taken as a reference, with hysteresis energies calculated as 0.0893, 0.1408, and 0.1937 J/mm^3^ for the PC/ABS, PC/ABS_20, and PC/ABS_80 specimens, respectively. Also, the hysteresis energies were associated with the energy absorption capability of the specimens at each loading/unloading cycle [59,61]. As seen from Figure 8d, the hysteresis energy increased with the addition of a PMMA component to the PC/ABS matrix, which indicated the fatigue life performance at each cycle or stress level. For instance, the PC/ABS_80 specimen has a higher hysteresis energy than both the PC/ABS and PC/ABS_20 specimens. This situation reveals that although the fatigue tests were not performed at the same stress amplitude (PC/ABS_80 was tested at the highest stress amplitude), the hysteresis energy value of the specimen exhibited a superior character. Therefore, it was an expected behavior that the PC/ABS_80 specimen exhibited superior fatigue life performance to both the PC/ABS and PC/ABS_20 samples, as seen in the fatigue life curves represented by the Basquin equation at low stress amplitude values.

The dynamic modulus of elasticity is determined as the slope of the stress–strain hysteresis curves at each cycle calculated from Equation (3). Also, the changes in the dynamic elastic modulus according to the loading/unloading cycles are given in Figure 8e. It can be found that the PC/ABS_80 specimen has a higher dynamic modulus than both the PC/ABS and PC/ABS_20 specimens. This situation revealed that the PC/ABS_80 specimen exhibits stiffer characteristics than the other ones. From Figure 8e, it can be said that the sudden decrease observed at the end of the loading cycles is a result of the stiffness loss of specimens. In order to examine the change in the dynamic elastic modulus more clearly, the changes in the dynamic elastic modulus normalized to the value at the 1000th cycle are given in Figure 8e for the PC/ABS, PC/ABS_20, and PC/ABS_80 specimens. It is observed that the increase in the normalized dynamic elasticity modulus does not exceed 5% depending on the number of cycles, but sudden decreases occur as the end of the number of cycles approaches, and the decrease in the PC/ABS (37%) and PC/ABS_20 (35%) specimens is higher than that of the PC/ABS_80 (%14) sample. This shows that the PC/ABS_80 specimen has superior stiffness and strain hardening ability [59,62].

The improvement in fatigue life performance with increasing PMMA content in the PC/ABS/PMMA ternary blends can be evaluated through the viscoelastic properties of the ternary blends. When the storage modulus of the PMMA and PC/ABS/PMMA specimens at room temperature was evaluated, it was determined that the storage modulus improves significantly compared to PC/ABS with an increasing PMMA ratio in the structure. It is known from previous studies that this situation occurs due to the interaction between hydrogen bond formations in the PMMA and ABS components in the structure and the decrease in molecular chain movement. [63,64]. It is also known that the energy absorption characteristics of the structure improve with an increasing storage modulus. In addition to this, the loop shifts, dynamic elastic modulus, and hysteresis energies during cyclic loading are related to structural rigidity and plastic deformation characteristics concerning the viscoelastic modulus [65]. Thus, it can be said that the improvement in the storage modulus with increasing PMMA content in the structure results in an improvement in fatigue life performance. When evaluated in this context, it can be concluded that blending PC/ABS with PMMA is an important alternative for obtaining the desired balanced properties specific to different applications, such as the impact strength, fatigue life, tensile strength, elastic modulus, and thermal stability. This situation shows that although PMMA exhibits lower fatigue life performance than all the samples at low and high stress amplitude values, mechanical properties and fatigue life behavior can be obtained within the optimum value range depending on the PMMA ratio in the blend structure.

## 5. Conclusions

This study reports on the effect of PMMA content on the chemical, thermomechanical, mechanical, and fatigue life properties of PC/ABS/PMMA ternary blends. First, PMMA, PC/ABS, and PC/ABS/PMMA ternary blends with different composition ratios were fabricated using the melt blending method. Then, their chemical and thermomechanical properties were assessed. The PMMA, PC/ABS, and PC/ABS/PMMA ternary blends were tested under tensile, impact, and fatigue loading conditions, and the effect of PMMA content was evaluated. In addition, the relationships between the chemical, viscoelastic, mechanical, and fatigue life properties were examined for the PMMA, PC/ABS, and PC/ABS/PMMA ternary blends at different composition ratios. Based on the experimental results, the following conclusions can be drawn:

- Analysis of the FTIR chemical behavior showed that the PC/ABS/PMMA ternary blend samples contained characteristic absorption peaks belonging to the three components constituting the structure. Also, it was determined that the peak intensity of the IR spectrum varied according to the PMMA composition ratio in the structure.
- The viscoelastic properties of the PMMA, PC/ABS, and PC/ABS/PMMA ternary blends were investigated using DMA analysis. This analysis indicates that the addition of PMMA to PC/ABS structures increased both the storage and loss modulus with increasing PMMA content. The peak value of the loss factor also increased with the increasing PMMA ratio in the ternary blends. Furthermore, the DMA results showed that blends with higher PMMA content exhibited a larger area under the loss modulus curve, reflecting greater structural energy absorption, while lower PMMA content resulted in a smaller area under the curve. This situation is an indicator of the improved energy absorption capability of the structure.
- For the PC/ABS specimen, mechanical properties such as the elastic modulus, yield strength, and ultimate tensile strength were found to be quite low compared to PMMA, while the elongation at break was significantly higher. Also, ternary blend specimens with the addition of PMMA to the structure have a higher elastic modulus, yield strength, and ultimate tensile strength with respect to the PC/ABS specimen. It is observed that with an increasing PMMA ratio in the structure, the elastic modulus, yield strength, and tensile strength values increase, while elongation-at-break values decrease. In the PC/ABS/PMMA ternary blend samples, it is observed that the impact strength decreases significantly with the increasing PMMA content in the structure.
- The experimental results from the fatigue tests demonstrate improvements in the life cycle performance of the PC/ABS/PMMA ternary blends at low and high stress amplitudes depending on the PMMA content. A 20% and 40% addition of PMMA to PC/ABS leads to an increase in fatigue strength and the absolute fatigue strength exponent, obtaining a high number of cycles at high stress amplitudes. Also, with higher PMMA content (60% and 80%), the life cycle performances improved at low stress amplitudes, resulting from the decrease in the absolute value of the fatigue strength exponents.
- The hysteresis energy values at different cycles increased with increasing PMMA content in the PC/ABS matrix, and the slope of the stress–strain hysteresis loops (dynamic modulus of elasticity) significantly improved with PMMA addition to the PC/ABS matrix. It was determined that the dynamic elastic modulus improved by approximately 12% to 58% with the addition of PMMA. Similarly, hysteresis energy values improved by approximately 26% to 117% due to the effect of PMMA.

In conclusion, the experimental results elucidate the complex relationship between PC/ABS/PMMA ternary blend contents and their characteristic properties, including their thermomechanical, mechanical, and fatigue life behaviors. The present findings contribute to a deeper understanding of the structure–property balance in PC/ABS/PMMA ternary blends, which is crucial for their application in areas such as the injection molding of lightweight automotive and aerospace components, which demands optimized performance. Future research will focus on investigating the tribological behavior of PC/ABS/PMMA ternary blends and identifying microstructural relationships.

## Figures and Tables

**Table 1:** Physical and mechanical properties of granules used in the injection-molding process of specimens.

Granules	Properties			
	Density (kg/m^3^)	Melt Flow Rate (g/10 min) (ISO 1133) [39]	Tensile Strength at Break (MPa) (ISO 527) [40]	Charpy Impact Strength (kJ/m^2^) (ISO 179/1) [41]
PC	1200	20	70	65
ABS	1060	4.0	50	14
PMMA	1190	4.2	78	1.4

**Table 2:** The absorption peaks and their functional groups observed in PC/ABS/PMMA ternary blends.

Wavenumber (cm^−1^)	Functional Group	Originate
~2990	C–H strength	PMMA and ABS
~2950–2850	C–H strength	PC and ABS
~2238	C≡N strength	ABS (acrylonitrile)
~1720	C=O strength	PMMA and PC (carbonyl)
~1600–1500	C=C aromatic	PC and ABS
~1450–1380	C–H bending	All components
~1260–1140	C–O strength	PMMA and PC
~752–700	Aromatic C–H bending	PC

**Table 3:** The glass transition temperatures of PMMA, PC/ABS, and PC/ABS/PMMA ternary blends.

Specimens	T_g,1_ (°C)	T_g,2_ (°C)	T_g,3_ (°C)
PMMA	114	-	-
PC/ABS	113	126	147
PC/ABS_20	117	-	-
PC/ABS_40	118	-	141
PC/ABS_60	115.6	-	143.7
PC/ABS_80	116.5	125	145

**Table 4:** The viscoelastic properties of PMMA, PC/ABS, and PC/ABS/PMMA ternary blends at 25 °C.

Specimens	Storage Modulus (MPa)	Loss Modulus (MPa)	Loss Factors
PMMA	3309	328	0.0991
PC/ABS	2245	84	0.0376
PC/ABS_20	2340	127.5	0.0531
PC/ABS_40	2490	161.7	0.0651
PC/ABS_60	2760	203	0.0742
PC/ABS_80	3280	268.3	0.0819

**Table 5:** Mechanical properties of PMMA, PC/ABS, and PC/ABS/PMMA ternary blend materials obtained from tensile tests.

Specimens	Mechanical Properties				
	Elastic Modulus (MPa)	Yield Strength (MPa)	Ultimate Tensile Strength (MPa)	Elongation to Break (%)	Impact Strength (J/m^2^)
PMMA	3314.7 ± 61.7	44.7 ± 0.49	54.5 ± 3.33	2.05 ± 0.28	3.8 ± 0.12
PC/ABS	2363 ± 50.6	33.3 ± 1.49	46.4 ± 1.28	16.47 ± 0.36	82.4 ± 1.3
PC/ABS_20	2383.7 ± 65.2	36.1 ± 1.53	52.2 ± 0.45	17.56 ± 0.36	33.4 ± 0.75
PC/ABS_40	2491.7 ± 41.6	37.5 ± 1.52	54.1 ± 1.58	10.35 ± 0.85	23.8 ± 0.64
PC/ABS_60	3068 ± 77.1	39.6 ± 0.66	61.8 ± 0.21	5.57 ± 0.97	18.4 ± 0.6
PC/ABS_80	3231 ± 75.7	42.4 ± 1.69	65.2 ± 2.1	3.79 ± 0.09	7.2 ± 0.41

**Table 6:** The number of cycles to failure data of PMMA, PC/ABS, and PC/ABS/PMMA ternary blends obtained from fatigue tests.

Specimens											
PMMA		PC/ABS		PC/ABS_20		PC/ABS_40		PC/ABS_60		PC/ABS_80	
Stress Amplitudes	Number of Cycles	Stress Amplitudes	Number of Cycles	Stress Amplitudes	Number of Cycles	Stress Amplitudes	Number of Cycles	Stress Amplitudes	Number of Cycles	Stress Amplitudes	Number of Cycles
10.8	3137 ± 726	12.8	7980 ± 725	14.3	5639 ± 375	15.2	4596 ± 627	13.8	5142 ± 508	16.2	3189 ± 352
8.2	16,837 ± 3538	9.8	20,871 ± 1052	10.9	13,813 ± 1691	11.6	11,277 ± 3275	10.6	19,886 ± 1134	12.4	9314 ± 493
6.9	48,004 ± 15,551	6.8	75,569 ± 5242	7.6	52,037 ± 3597	8.0	33,931 ± 2624	7.3	50,167 ± 10,962	8.6	30,406 ± 3520
5.7	126,304 ± 7365	5.3	151,605 ± 6107	5.9	85,254 ± 3568	6.2	87,337 ± 3095	5.7	125,692 ± 17,785	6.7	80,821 ± 7860

## Data Availability

The original contributions presented in this study are included in the article. Further inquiries can be directed to the corresponding author.
